# Foreign Body Reaction to Ion-Beam-Treated Polyurethane Implant

**DOI:** 10.3390/ma17153833

**Published:** 2024-08-02

**Authors:** Vyacheslav S. Chudinov, Igor N. Shardakov, Valery V. Litvinov, Sergey Y. Solodnikov, Elena Y. Chudinova, Irina V. Kondyurina, Alexey V. Kondyurin

**Affiliations:** 1 Institute of Continuous Media Mechanics, Ural Branch, Russian Academy of Sciences, Perm 614013, Russia; chudinovsl@mail.ru (V.S.C.); shardakov@icmm.ru (I.N.S.); 2 Therapeutic Faculty, Perm State Medical University, Perm 614990, Russia; drlitvinov@mail.ru; 3 Applied Chemical and Biochemical Research Center, Perm National Research Polytechnic, Perm 614990, Russia; s.u.solodnikov@rambler.ru; 4 Imbiocom Ltd., Perm 614990, Russia; elen_shich@mail.ru; 5 School of Medicine, University of Sydney, Camperdown, NSW 2050, Australia; i.kondyurina@gmail.com; 6 Ewingar Scientific, Ewingar, NSW 2469, Australia; 7 School of Physics, University of Sydney, Camperdown, NSW 2050, Australia

**Keywords:** foreign body reaction, biocompatibility, implant, polyurethane, ion beam treatment

## Abstract

All artificial materials used for implantation into an organism cause a foreign body reaction. This is an obstacle for a number of medical technologies. In this work, we investigated the effect of high-energy ion bombardment on polyurethane for medical purposes and the reaction of body tissues to its insertion into the mouse organism. An analysis of the cellular response and shell thickness near the implant showed a decrease in the foreign body reaction for implants treated with high-energy ions compared to untreated implants. The decrease in the reaction is associated with the activation of the polyurethane surface due to the formation on the surface layer of condensed aromatic clusters with unbonded valences on the carbon atoms at the edges of such clusters and the covalent attachment of the organism’s own proteins to the activated surface of the implant. Thus, immune cells do not identify the implant surface coated with its own proteins as a foreign body. The deactivation of free valences at the edges of aromatic structures due to the storage of the treated implant before surgery reduces surface activity and partially restores the foreign body response. For the greatest effect in eliminating a foreign body reaction, it is recommended to perform the operation immediately after treating the implant with high-energy ions, with minimal contact of the treated surface with any materials.

## 1. Introduction

Modern artificial implants are used to save lives or to improve the quality of life for patients. Implants such as pacemakers, implantable cardio defibrillators, ventricular assist devices, heart valves, blood vessels, and catheters made of polyethylene, polypropylene, polytetrafluorethylene, polyamide, polyethylene terephthalate, and polydimethylsiloxane; cochlear implants, staples, and nasal implants made of polydimethylsiloxane, parylene, and polyethylene; penile implants, Foley implants, urinary sphincters, hernia or vaginal meshes made of polydimethylsiloxane, polyethylene, polytetrafluorethylene, polyamide, and polypropylene; breast implants, cheek, jaw and chin implants, lip implants, and hip implants made of polypropylene, polyethylene terephthalate, polytetrafluorethylene, and polydimethylsiloxane; intrauterine devices, intravaginal rings, urogynecologic mesh implants, and fetal micro-pacemakers made of polydimethylsiloxane, polypropylene, and polyurethane; retinal prostheses, intraocular lenses, glaucoma valves, orbital implants, and ophthalmic implants made of polymethylmethacrylate, polyethylene, polytetrafluorethylene, and polyamide, and other devices are used in surgery rooms [1,2,3,4,5,6,7,8,9,10]. These materials have been tested and approved by the FDA as providing an acceptable level of cytotoxicity, sensitization, irritation, acute systematic toxicity, sub-chronic toxicity, genotoxicity, hemocompatibility, chronic toxicity, carcinogenicity, reproductive development, and biodegradation. The most stringent requirements are applied to the materials used for long-term implants (more than 30 days of an implant in an organism is considered permanent) and sensitive organs such as ophthalmic devices. Materials for implants are selected based on conditions in the organism such as mechanical loading and movement, pH environment, and aggressive liquids, and on the functionality of the implant such as electro-conductivity or electro-resistivity, mechanical strength, softness and elasticity, multiple cyclic loads, low or high friction, required biodegradation rate, and corrosiveness. The product of biodegradation must satisfy the biocompatibility requirements, too. Also, the materials for the implant must be sterilizable by certified methods such as ethylene oxide, steam, UV irradiation, or gamma irradiation [11,12,13,14,15,16].

However, all materials inserted into an organism’s tissue cause an immune response of the organism called a foreign body reaction. The result of a foreign body reaction is an acute and chronic inflammatory response, pain, discomfort, and an isolation of the implant from the organism tissue by a collagen shell. In some cases, the isolation of an implant due to a foreign body reaction can support a bacterial infection. In the worst-case scenario, the organism’s reaction can impair the functionality of the implant. In most cases, immunodepression therapy can decrease the activity of the organism reaction. However, in this case, the organism becomes unprotected against infections. For some patients, immunodepression therapy is not allowed and implant surgery is problematic [17,18,19,20,21,22,23,24,25,26,27,28].

The foreign body reaction in response to the artificial implant is based on the adsorption of host proteins on the surface of the implant and the change in protein conformation. The proteins with a changed conformation are detected by immune cells, which activate the immune reaction. Because the proteins adjust their conformation to any implant material, the immune reaction is observed for all materials of implants [29,30,31,32,33,34].

The decrease or absence of the foreign body reaction was observed in a number of our studies with an ion-beam-treated polymer surface [35,36,37]. The surface of the polymer implant was treated by high-energy ions, then the implant surface became carbonized [38] with the presence of free radicals at the edges of condensed aromatic structures. These free radicals provide the high surface energy of the implant surface and strong covalent bonding of the adsorbed proteins [39]. As a result, the implant surface is covered by host proteins with native conformations, which are strongly linked to the implant surface. The protein coverage of the implant surface is total [36]. Therefore, the immune cells do not contact proteins with changed conformations and are not activated.

However, the possibility of the absence of the foreign body reaction is still in doubt for many researchers. In this paper, we present the results of subsequent studies with medically approved polyurethane and discuss the results on the absence of the foreign body reaction.

## 2. Materials and Methods

Polyurethane SKU-PFL, certified for long-term implants in the human body, was used [40,41,42]. Polyurethane was synthesized from polytetrahydrofuran with terminal hydroxyl groups terminated by toluene diisocyanate. Aromatic diamine was used for curing. Polyurethane films 0.5 mm-thick were synthesized for the study. Before use, the polyurethane films were swollen in toluene to wash out unreacted products and then the films were dried until the solvent was completely removed. The presence of solvent and unreacted products was monitored by FTIR ATR spectra and mass loss measurements. The final polyurethane was completely 3D crosslinked.

In this work, we used plasma immersion ion implantation (PIII) as one of the ion beam treatment methods. In PIII, the ion beam is formed directly near the surface of the sample being treated, while in the ion beam treatment method, the beam is usually formed at some distance from the sample. For polymers, the direct use of the PIII method is not applicable, because the electric charge accumulated in the polymer during treatment does not flow to the electrode, as in the case of metals, and affects the formation of the ion beam. To eliminate this influence, the polymer is placed in a Faraday cap, which means the polymer is covered with a metal mesh connected to the electrode. Thus, the charge accumulated on the polymer does not affect the ion beam formation above the mesh surface. Therefore, this is not a classical plasma immersion method. Also, it should be noted that while the term PIII is accepted in the literature for the treatment of polymers, in fact, low fluences of up to 10^16^ ions/cm^2^ are usually used for polymers and, accordingly, the implantation mode, when the concentration of incoming ions is comparable to the concentration of polymer atoms, is not achieved. Therefore, we use the term PIII in this work to denote the features of the plasma system following the established terminology, although in fact we are using ion beam treatment of the polymer.

Polyurethane samples in the form of disks with a diameter of 100 mm and a thickness of 0.5 mm were treated on both sides with 20 keV energy nitrogen ions. The plasma immersion ion implantation method was used. The treatment was conducted in a vacuum chamber equipped with rotary and turbomolecular pumps. The maximum pressure of the residual atmosphere was about 10^−3^ Pa. The working pressure of nitrogen gas was 0.27 Pa. The plasma in the chamber was ignited from a radio frequency (13.75 MHz) generator. The power of the absorbed radio frequency energy was controlled by the matching box and managed in the 85–90 Watt range. A high negative voltage pulse was applied to the electrode on which the polyurethane films were fixed and covered with a stainless-steel mesh connected to the electrode. The fluence of the ion treatment was measured from the absorption spectra of the satellite polyethylene film treated under the same conditions according to the method described in [36]. The specific fluence of 1.25 × 10^13^ ions/(cm^2^·s) was found. The treatment of polyurethane samples was carried out at the University of Sydney. After the treatment, the samples were packaged in plastic films and transported to Perm University, where they were stored at room temperature until the experiment with animals. The total storage time of the treated samples from the ion treatment to the operation was 2 months.

The electron spin resonance (ESR) spectra of the treated and untreated polyurethanes were recorded on a SpinScan X spectrometer (ADANI, Minsk, Belorussia) at room temperature 25 °C in a quartz capillary. The polyurethane film was tightly rolled into tubes and placed in a capillary. The capillary was installed in the sample holder in the cavity between the spectrometer magnets. The spectra were recorded at a magnetic field central position of 333.835070044611 mT. The magnetic field variation range was 25 mT. The modulation frequency was 0.1 GHz with a modulation amplitude of 300 μT. The electromagnetic field frequency was 9.362794 GHz. The number of scanning points was 1000. The signal was accumulated from 60 to 240 s, dependent on noise level. Spectra were analyzed using eSpinoza software developed for the ADANI SpinScan X spectrometer.

The polyurethanes were measured using a Dimension Icon atomic force microscope (Veeco, Plainview, NY, USA). The PeakForce nanomechanical mapping mode with a ScanAssyst-Air probe (0.4 N/m) was used. The images of 12 × 12 μm^2^ in size were obtained. Topography and maps of mechanical properties of the surface were analyzed. Relief images were obtained by combining the color scale and surface lighting. The surfaces were levelled by an inclined plane.

A Digilab spectrometer (Agilent, Mulgrave, VIC, Australia) with a spectral resolution of 4 cm^−1^ and a number of scans of 500 was used for Fourier transform infrared (FTIR) attenuated total reflection (ATR) spectra. A Harrick ATR attachment (Pleasantville, NY, USA) with a germanium crystal with a 1 × 5 cm^2^ sample area and a light beam incidence angle of 45 degrees was used. The spectral software OPUS 7, Resolution Pro 4.0, and Galactic Grams 7.01 were used for analysis. To exclude the spectrum of water vapor, the spectrum of residual water vapor was separately recorded in the spectrometer and subtracted from the spectrum of polyurethane with normalization coefficients, which were selected for each spectrum to completely subtract the spectrum of water. The linear baseline and subtraction of spectra with a normalization coefficient was used to analyze spectral line intensity.

A cell culture of human aortic endothelial cells (HCAECs) was used to evaluate the adsorption of cells onto the surface and the cytotoxicity of polyurethane. The samples were sterilized with ultraviolet light for 10 min on both sides of the sample before applying the cell culture. After sterilization, all samples were kept in a fume hood with sterile reverse airflow. The samples were placed on the bottom of 24-well plates and cells were seeded onto them. MesoEndo Endothelial Cell Media was used. The initial concentration of the cells was 2 × 10^4^ cells/mm^2^. The cells were proliferated in a sterile incubator at 37 °C in an atmosphere of 5% CO_2_. The cells were incubated for 2 and 5 days. Then, the cells were fixed with an 80% ethyl alcohol solution for 30 min. The fluorescent dye phalloidin (Phalloidin-TRITC) was used to stain actin microfilaments. Immediately before the observation, the cells were stained with the fluorescent dye DAPI to visualize cell nuclei. After staining, the samples were placed between two glass slides. An Olympus (Tokyo, Japan) IX71 microscope with a digital camera was used to observe the stained cells. Each sample was photographed 10 times in a blind manner using a 10× lens. Detailed photographs of the spread-out cells were taken with a 20× lens. Statistical analysis of the images was carried out using the ImageJ 1.52a software. The high-resolution images of actin microfilaments and nuclei were combined.

In animal trial experiments, the polyurethane samples were used in the form of flat disks with a diameter of 8 mm. Polyurethane samples were implanted subcutaneously into six certified SD (Sprague Dawley) laboratory rats. The age of the animals at the time of the experiment was 3 months; the period of development and growth was completed. Only males were used in the experiment to exclude background hormonal changes. The animals were kept under standard laboratory conditions: temperature 20–22 °C and humidity 45–60%. Feeding of the animals was organized with certified food for laboratory animals (CHARA, Assortment-Agro Ltd., Sergiev Posad, Russia). When caring for the animals, certified bedding (REHOFIX, Rosenberg, Germany) was used. The animal study protocol was approved by the Ethics Committee of Institute of Ecology and Genetics of Microorganisms of Russian Academy of Science, protocol number 3 from 30 November 2015.

Five groups of polyurethane samples were prepared: untreated and treated by the ions with fluences (treatment time) of 5 × 10^14^ ions/cm^2^ (40 s), 10^15^ ions/cm^2^ (80 s), 5 × 10^15^ ions/cm^2^ (400 s) and 10^16^ ions/cm^2^ (800 s). Five samples were implanted into each animal, one from each group. The samples in the animals were exposed for 1 month and 5 months, for 3 animals each. The operations were performed under general anesthesia at the rate of 0.3 mL of the anesthetic Zoletil-50 per animal (Fort Worth, TX, USA). No other drugs were applied.

Each polyurethane sample was placed subcutaneously between the fascia in a separate place on the back of the animal. The distance between the samples was 5–8 mm. Suture Vicryl 4/0 was used for stitching. One suture was placed per one cut. Ethyl alcohol was used to sterilize the incision site before surgery. After surgery, the suture was treated once with 3% hydrogen peroxide. The implant was placed in the pocket so that the suture was offset along the surface of the skin relative to the sample, in order to avoid contact with the antiseptic.

After surgery, each animal was placed in a separate cage. Periodic condition monitoring was carried out and observations were recorded based on a standard animal-condition-monitoring protocol.

Euthanasia was carried out using carbon dioxide in accordance with the rules of the European Convention for the Protection of Vertebrate Animals Used for Experimental or Other Scientific Purposes. The implant samples were cut out along with the tissue, approximately 3.5 mm around the polymer sample. The extracted tissue pieces were fixed in a 10% neutral formaldehyde solution for 24 h. All samples were stored in a closed container in the refrigerator before histological investigation.

Implant samples with surrounding tissues were dehydrated, degreased, and embedded in paraffin according to the generally accepted method [43]. Sections 5 μm-thick were prepared from paraffin blocks and stained with hematoxylin and eosin. To stain microslides, dye solutions were used, prepared in accordance with the recommendations for histological techniques [44]. Viewing and photographing of microslides was carried out on an Axioimager A1 microscope (ZEISS, Oberkochen, Germany) equipped with an Axiocam MRc5 digital camera (ZEISS, Germany) and a Hirox KH-7700 digital optical 3D microscope (HIROX, Tokyo, Japan). The number and ratio of the main cellular elements (segmented nuclear neutrophils, eosinophils, macrophages, fibroblasts, lymphocytes, and plasma and mast cells), collagen, and elastic fibers, as well as the thickness and nature of the capsule formed around the implant were assessed.

The ImageJ software was used for morphometric analysis and to calculate the size of the connective tissue shell. Then, the thickness of the shell was calculated as the ratio of the capsule area to the length of the implant–organism interaction contour. The contour of the capsule for calculating the area and length was selected manually using the ROI (region of interest) Manager tool of ImageJ software. The thickness and number of cells were calculated for 2–3 sections of one tissue sample. Statistical processing of the obtained data and plotting of graphs were carried out using the RStudio v.1.2 program. The Student, Tukey, and Student–Newman–Keuls tests, as well as the statistical analysis of variance (ANOVA) were applied to the obtained data. The applicability of Student’s *t*-test, ANOVA, Tukey’s range test [45], and the Student–Newman–Keuls test was determined by checking the normality of the distribution of the sample data using the Shapiro–Wilk test and the homogeneity of variances using the Levene test [46]. To satisfy the conditions of a normal distribution of samples and a homogeneity of variances in the distribution density of neutrophils and eosinophils, the data set values were converted to logarithms as follows:

$$x^{\prime }=log(a+x)$$

where *x* is the initial value, *x*′ is a new value, and *a* is a fitting coefficient [47,48].

## 3. Results

### 3.1. Surface Structure of Polyurethane

**Figure 1:**
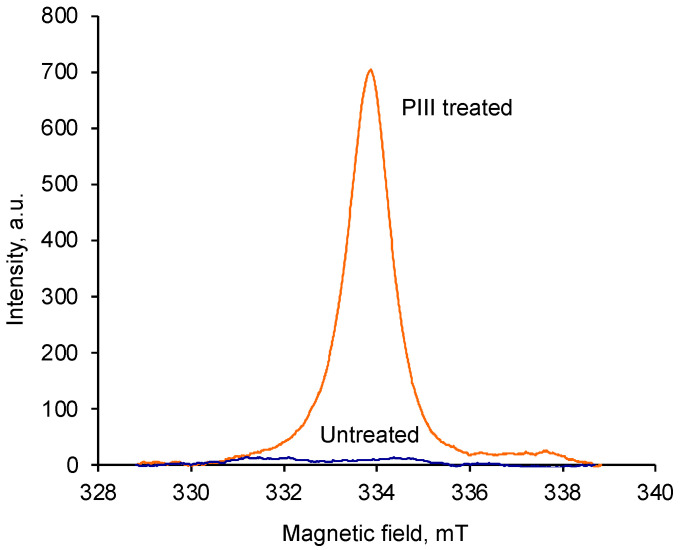
Electron spin resonance spectra of polyurethane, both untreated and treated with a fluence of 5 × 10^15^ ion/cm^2^. The spectra were integrated, and the intensity is proportional to the concentration of free radicals in polyurethane.

The spectrum of the untreated sample shows a low-intensity signal caused by free radicals in polyurethane as a result of degradation under environmental conditions (Figure 1). The treated sample shows a high-intensity signal caused by free radicals generated after the ion beam treatment. The spectrum was recorded for the treated samples stored for 2.5 months under laboratory conditions.

The g-factor (2.0028) of the ESR signal for the treated polyurethane is close to the g-factor of free electrons (2.0025) and corresponds to the g-factor of unpaired free electrons delocalized on graphite structures. Such unpaired electrons can be located on carbon atoms at the edges of condensed aromatic clusters. Typically, free radicals have a short lifetime at room temperature due to their high activity and quickly react with nearby molecules—proton donors or unsaturated structures. The stable signal in the treated polyurethane film can be explained by the delocalization of the unpaired electron on the π electrons of the graphite structure. In this case, the free radical cannot react with nearby molecules and remains in the treated film for a long time.

**Figure 2:**
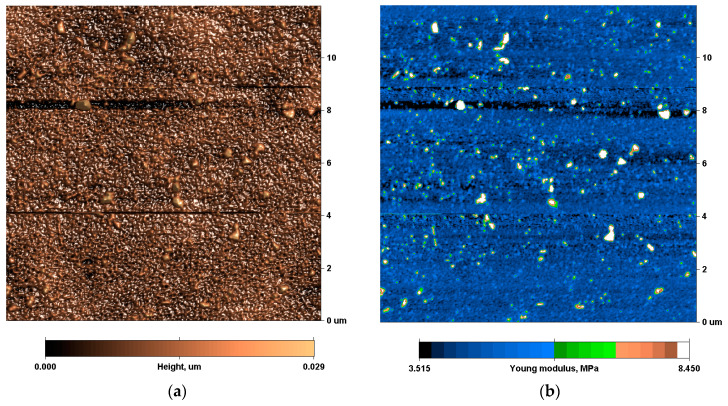

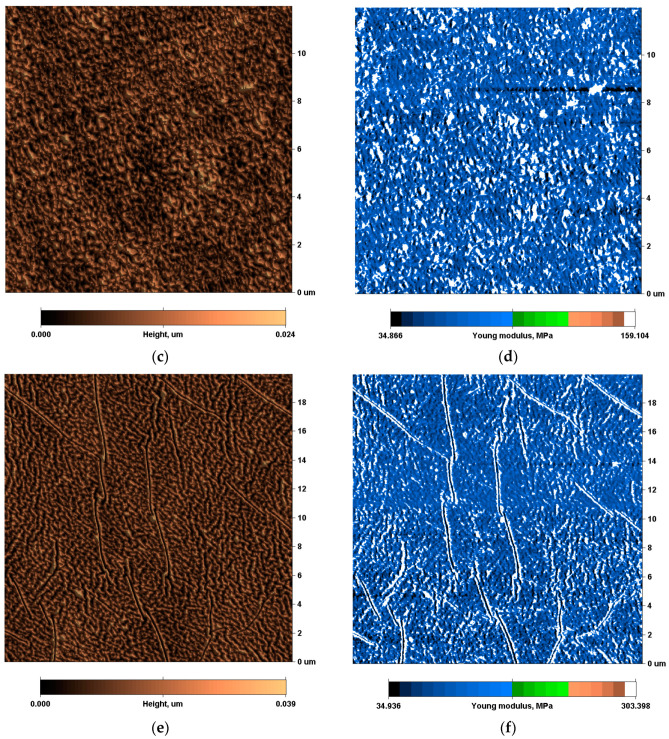

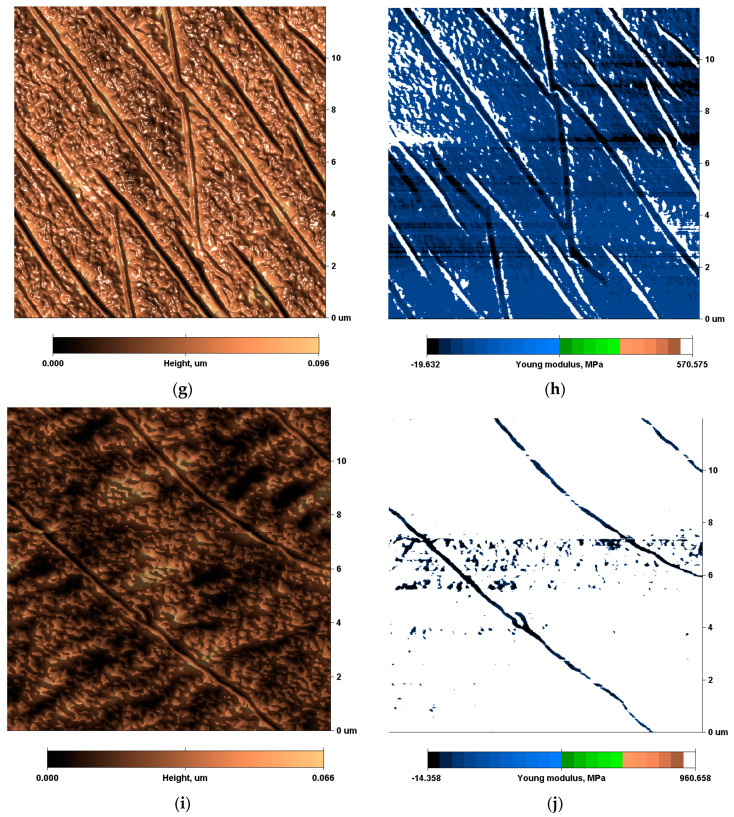
Topography (**a**,**c**,**e**,**g**,**i**) and Young’s modulus (**b**,**d**,**f**,**h**,**j**) of polyurethane surface, untreated (**a**,**b**) and treated with fluence of 5·10^14^ ions/cm^2^ (**c**,**d**), 10^15^ ions/cm^2^ (**e**,**f**), 5·10^15^ ions/cm^2^ (**g**,**h**), and 10^16^ ions/cm^2^ (**i**,**j**).

The surface of the untreated polyurethane was quite smooth and was determined by the morphology of the substrate on which the polyurethane was synthesized (Figure 2). The surface relief of the treated samples was characterized by cracks and waves. The cracks became visible in samples treated 80 s and longer. The modulus of the surface layer increased with the treatment time. At 800 s, the surface modulus became almost 1 GPa. The bottom of the cracks had a low modulus. The topography and modulus distribution of the treated polyurethane surface corresponded to a hard surface layer on the top. Due to internal stresses, the top hard layer was cracked, and the layer of the untreated bulk polyurethane underneath became visible.

**Figure 3:**
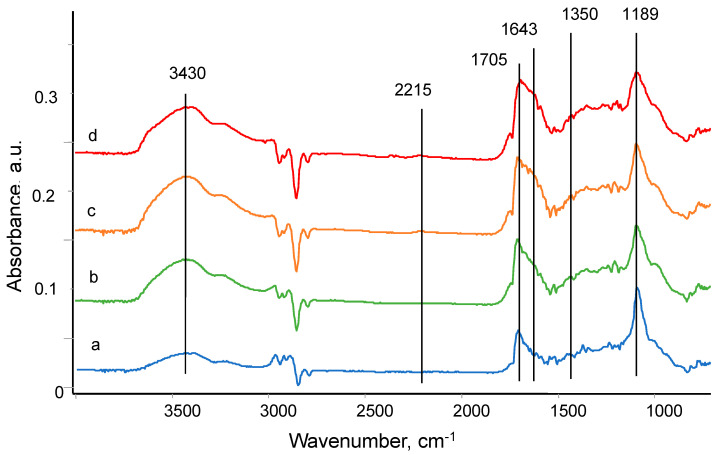
FTIR ATR spectra of polyurethane treated by PIII with fluence of 5 × 10^14^ ions/cm^2^ (a), 10^15^ ions/cm^2^ (b), 5 × 10^15^ ions/cm^2^ (c), and 10^16^ ions/cm^2^ (d). The spectrum of untreated polyurethane is subtracted from each spectrum of treated polyurethane.

The differential spectra of the treated polyurethane have wide vibration lines of hydroxyl groups in the region of 3600–3200 cm^−1^ with a maximum of 3430 cm^−1^; a nitrile group line at 2215 cm^−1^; a wide line with a multiplet structure in the region of 1750–1500 cm^−1^ attributed to vibrations of the double bonds C=O, C=N, and C=C; a wide continuum in the region of 1350–1200 cm^−1^ attributed to vibrations of C-C bonds; and an intense line at 1189 cm^−1^ attributed to vibrations of C–O bonds (Figure 3). The significantly greater width of the new lines compared to the lines of the original polyurethane indicated a disordered structure that appeared as a result of the treatment with ions. Therefore, the surface of the treated polyurethane consisted of new chemical structures associated with the carbonization, oxidation, and depolymerization of the polyurethane macromolecules. The spectra of the bulk layers of polyurethane and untreated side do not show changes.

**Figure 4:**
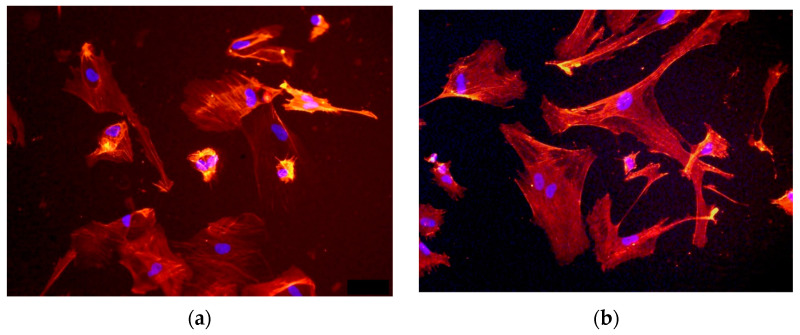
Endothelial cells attached to (**a**) untreated and (**b**) treated (5 × 10^15^ ions/cm^2^) polyurethane surface and proliferated for 5 days. Size of images is 640 × 480 μm.

Human aortic endothelial cells were used to evaluate the adsorption of cells onto the surface and the cytotoxicity of treated and untreated polyurethane. When the endothelial cells were seeded onto the polyurethane surface, the cells were distributed evenly over the entire surface of the sample. Within two hours, the cells settled on the surface of the untreated polyurethane, but the cells retained the compact shape inherent to cells in solution. Two days after seeding, the cells maintained a compact shape and formed cell clusters with adjacent cells. A small proportion of the cells adhered to the surface of the untreated polyurethane, forming a spindle shape, but the majority of the cells remained in a compact state. After 5 days of proliferation, some of the cells spread out on the surface of the untreated polyurethane, but most of them remained in a spindle-shaped state (Figure 4). The number of cells on the untreated polyurethane increased and reached the values recorded on the control TCP plastic. This result showed an absence of cytotoxicity on untreated polyurethane.

When the cells were seeded onto the surface of the treated polyurethane, they were also distributed evenly over the entire surface and were anchored on the surface. Some of them formed a spindle shape after 2 h. After 2 days, most of the cells were well spread over the surface of the polyurethane. The distribution of cells over the surface of the treated polyurethane did not depend on the treatment fluence. Within 5 days of proliferation, most of the surface of the treated polyurethane became covered with spread cells. In this case, the spreading of cells and the density of their distribution over the surface of the treated samples were practically independent of the treatment fluence. A similar pattern of cell spreading was observed for the control plastic. This result also showed an absence of cytotoxicity on the treated polyurethane.

### 3.2. Reaction on Untreated Implants

The results of monitoring the condition of the animals showed that there were no changes in motor activity or behavioral reactions. There were no deviations in food or water intake. Body weight did not change significantly. No disorders of the mucous membranes, skin, or hair were observed. Thus, the polyurethane samples implanted for a long time did not affect the general condition of the animals.

**Figure 5:**
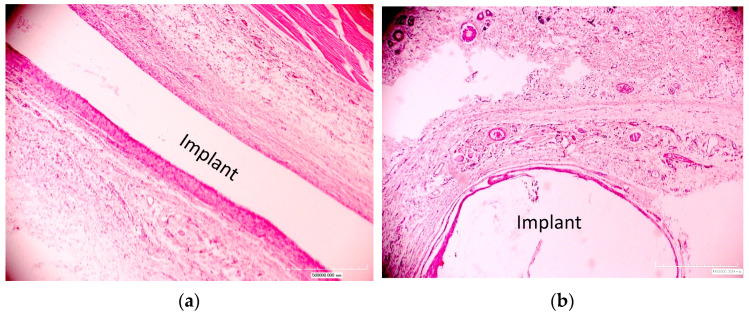
Micrographs of histological tissue sections stained with hematoxylin and eosin. Exposure period of the polyurethane implant in the body is 1 month. (**a**) Untreated and (**b**) treated with fluence of 5 × 10^15^ ion/cm^2^. The position of the implant is indicated. The image size is 2.2 × 1.65 mm.

**Figure 6:**
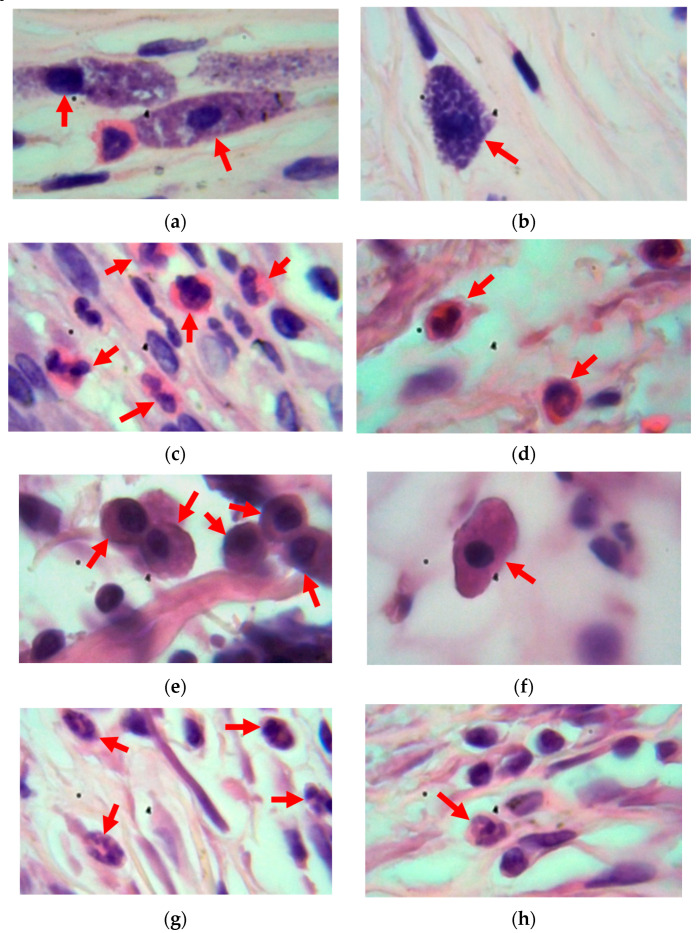
Characteristic view of specific cells in the tissue near the untreated (**a**,**c**,**e**,**g**) and ion-beam-treated (**b**,**d**,**f**,**h**) implants. Arrows shows specific cells: (**a**,**b**) is basophils, (**c**,**d**) is eosinophils, (**e**,**f**) is macrophages, and (**g**,h) is neutrophils.

The analysis of the cellular and tissue response for the untreated polyurethane in all animals showed a moderate foreign body reaction, as is standard for modern medical-grade materials. Around the untreated implants, the formation of granulation tissue with inflammatory cell infiltration and the formation of a dense connective tissue shell of fibroblasts and collagen fibers was observed (Figure 5). There was a moderate increase in the number of eosinophils, neutrophils, macrophages, lymphocytes, plasma cells and basophils in the connective tissue shell area compared to tissue areas more distant from the implant (Figure 6). There were large macrophages, including those exhibiting the phenomenon of phagocytosis. Multinucleated cells were observed around the shell and at the site of interaction of the soft tissue with the implant. On the side of the deeper tissues of the body, the membrane was thicker and denser, with more fibrous tissue and collagen fibers than on the side of the epidermis. One could also note the presence of a larger number of cellular elements, as well as greater fibroblast activity.

In general, along the edge of the entire shell in places adjacent to the surrounding tissues, accumulations of the blood cells were clearly visible: neutrophils, eosinophils, plasmacytes, and lymphocytes. Mast cells were also located in the thickness of the connective tissue shell. An uneven distribution of cells was observed: in places where the membrane was denser and thicker, there were fewer cellular elements. Where the membrane was looser and thinner, there were accumulations of eosinophils, neutrophils, and lymphocytes. Also, a small number of newly built vessels could be seen near the implant.

**Figure 7:**
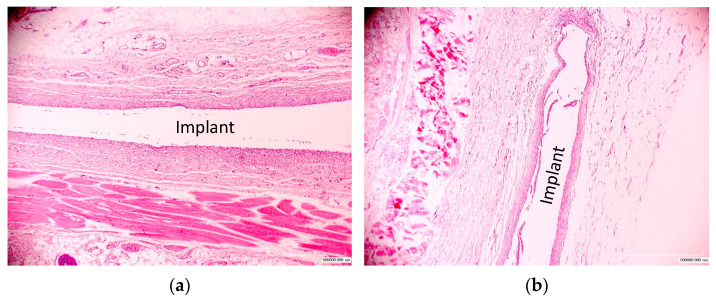
Micrographs of histological tissue sections stained with hematoxylin and eosin. Exposure period of the polyurethane implant in the body is 5 months. (**a**) Untreated and (**b**) treated with fluence of 5 × 10^15^ ion/cm^2^. The position of the implant is indicated. The image size is 2.2 × 1.65 mm.

When the implants were exposed for 5 months, a characteristic chronic reaction with a low infiltration of cellular elements and the absence of pronounced fibrosis was observed (Figure 7). A moderately increased number of eosinophils, neutrophils, macrophages, lymphocytes, and plasma and basophil mast cells remained in the connective tissue shell and in the area adjacent to the surrounding tissues compared to tissue areas more distant from the implant.

### 3.3. Reaction on Treated Implants

The general visual analysis showed a significant difference in the tissue around the treated implants compared to the tissue around the untreated implants. Around the treated polyurethane samples, the formation of a thinner and looser connective tissue shell without pronounced fibrosis with minimal inflammatory infiltration was observed (Figure 5 and Figure 7). The distribution of cells was approximately equal in the area close to the implant and in the surrounding deeper tissues. In some areas, there were small accumulations of neutrophils and eosinophils closer to the connective tissue shell.

Around the implants treated with a fluence of 10^15^ ions/cm^2^, the cells were localized in clusters in the form of weak granulation tissue. There were fewer cellular elements in the connective tissue shell than at its edges. Closer to the epidermis, the shell was denser with a large number of collagen fibers. Around the implants treated with a fluence of 5 × 10^15^ ions/cm^2^, the resulting connective tissue shell contained a significant number of fibers and fibroblasts. The remaining cellular elements were predominantly located in small clusters at the junction of the connective tissue shell with the surrounding tissues. The reaction to implants treated with a fluence of 10^16^ ions/cm^2^ was characterized by the arrangement of cells in clusters along the edge, and many fibroblasts and collagen fibers around the connective tissue shell. No differences were found in the immune cells themselves located near the untreated and treated implants (Figure 6).

### 3.4. Statistical Analysis of Cellular and Tissue Response

For the quantitative measurements, eosinophil and neutrophil cells were identified and counted for each sample. The number of eosinophils and neutrophils in the connective tissue shell was calculated per 1 mm of the length of the contour of the implant–organism interface in the section, and per 1 mm^2^ of the area of the connective tissue shell for the distribution density of eosinophils and neutrophils. This double analysis was caused by the uneven distribution of immune cells in tissues at different distances from the implant surface. Thus, a statistical analysis was performed consisting of four quantitative characteristics of the cellular response.

The results of the averaged data for all samples showed that the number of eosinophils per 1 mm of the length of the contour of the implant–organism interface and the density of their distribution in the tissue near the treated implants were less than those near the untreated samples by 5 to 12 times depending on the fluence of the treatment. The number of neutrophils per 1 mm of the length of the implant–organism interface contour and the density of their distribution for the treated implants were less than those for the untreated samples by 2 to 4 times depending on the fluence of the ion plasma treatment. These results showed that the most promising regime of the treatment to minimize the immune cellular reaction was with a fluence of 10^16^ ions/cm^2^ (Table 1). However, the thickness of the shell around the implants treated with this fluence after 5 months of exposure in the organism was not the lowest among all the other treatment fluences (Table 2). This can be explained by the formation of a rigid surface layer and the features of its relief, such as waves and cracks, which can deform or even injure adjacent tissues at high treatment fluences. In addition, through the cracks, proteins and immune cells can reach untreated areas of the polyurethane and cause an immune foreign body reaction in these areas.

The obtained data on the cellular response were analyzed using mathematical statistics using the Tukey test, ANOVA, and the Student–Newman–Keuls method. The diagrams representing the results of the data analysis according to the Tukey test contain the following designations: * corresponds to a significance of α = 0.05, ** corresponds to a significance of α = 0.01, *** corresponds to a significance of α = 0.001, and “n/s” corresponds to an indistinguishable difference between the values at the significance level of α > 0.05. In the statistical tests described below, the calculated values of the statistical criteria are compared with the critical value at the α significance level. This means that the probability of accepting the hypothesis of the equality of sample means, the so-called *p*-value, is less than the critical value of the significance level α.

**Figure 8:**
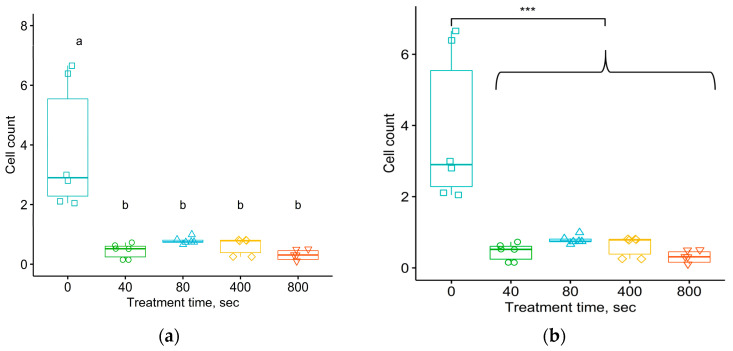
The number of eosinophils in the connective tissue shell per 1 mm of the length of the contour of the implant–organism interface, around implants treated at different fluences. Comparison of sample means using Student–Newman–Keuls (**a**) and Tukey (**b**) tests. ANOVA test shows *p* = 4.5 × 10^−9^. For Student–Newman–Keuls test, the “a” and “b” notifications are related to the groups of data with significant differences in means between them.

**Figure 9:**
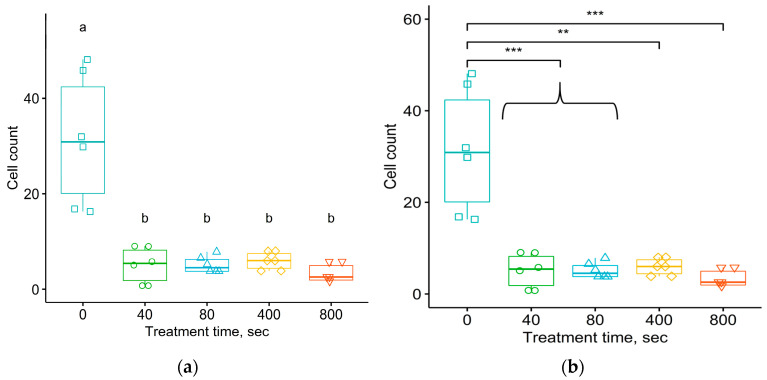
The number of eosinophils per 1 mm^2^ area of the connective tissue shell around implants treated at different fluences. Comparison of sample means using Student–Newman–Keuls (**a**) and Tukey (**b**) tests. ANOVA test shows *p* = 1.01 × 10^−5^. For Student–Newman–Keuls test, the “a” and “b” notifications are related to the groups of data with significant differences in means between them.

The ANOVA dispersion test was used to show differences in the average numbers of eosinophils in the connective tissue shell per 1 mm of the length of the implant–organism interface in the section (Figure 8) and their distribution density (Figure 9) with a *p*-value of 4.5 × 10^−9^ and 10^−5^, respectively. The Student–Newman–Keuls test was used to show differences between the average values of the number of eosinophils in the connective tissue shell per 1 mm of the length of the implant–organism interface for the untreated samples in comparison with the treated ones, and between the distribution densities of eosinophils per 1 mm^2^ area of the connective tissue shell for the untreated samples versus the treated samples.

The significance of the differences was confirmed by the Tukey test, the *p*-values of which, when comparing the untreated samples with the four groups of treated ones, corresponded to the critical level of significance (Figure 8 and Figure 9). For the average numbers of eosinophils in the connective tissue shell per 1 mm of the length of the contour of the implant–organism interface, the *p*-value range was from 10^−7^ to 2.7 × 10^−6^. For the distribution density of eosinophils per 1 mm^2^ of the area of the connective tissue shell, the *p*-values ranged from 1.45 × 10^−5^ to 1.3 × 10^−3^.

Therefore, a comparison of the eosinophil mean data for the untreated and treated samples using the Student–Newman–Keuls and Tukey tests revealed a statistically significant difference. However, no statistically significant difference was found at the significance level for the eosinophil mean data for groups of samples treated with different fluences.

**Figure 10:**
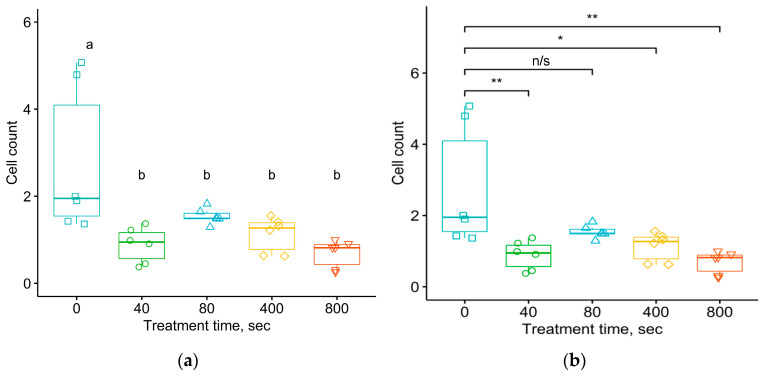
The number of neutrophils in the connective tissue shell per 1 mm of the length of the contour of the implant–organism interface, around implants treated at different fluences. Comparison of sample means using Student–Newman–Keuls (**a**) and Tukey (**b**) tests. ANOVA test shows *p* = 0.00149. For Student–Newman–Keuls test, the “a” and “b” notifications are related to the groups of data with significant differences in means between them.

**Figure 11:**
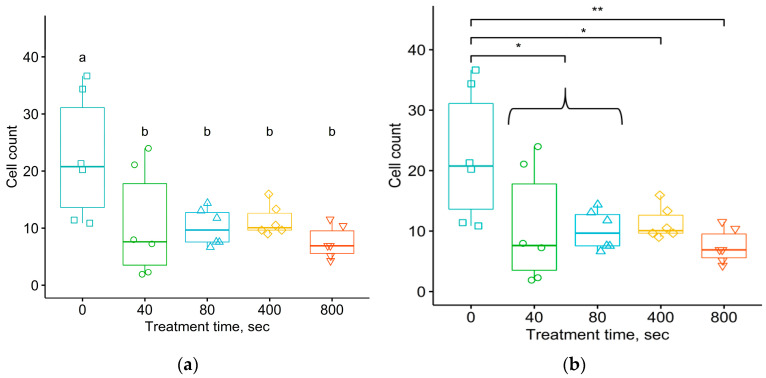
The number of neutrophils per 1 mm^2^ area of the connective tissue shell around implants treated at different fluences. Comparison of sample means using Student–Newman–Keuls (**a**) and Tukey (**b**) tests. ANOVA test shows *p* = 0.00956. For Student–Newman–Keuls test, the “a” and “b” notifications are related to the groups of data with significant differences in means between them.

Figure 10 and Figure 11 show the results of similar statistical analyses of data for the number of neutrophils in the connective tissue shell formed around the implant after one month in the organism. The ANOVA analysis of variance of the average values of the number of neutrophils in the connective tissue shell per 1 mm of the length of the contour of the implant–organism interface and their distribution density for samples of untreated and treated samples with different fluences shows a significant difference with *p*-values of 1.49 × 10^−3^ and 9.6 × 10^−3^ respectively.

These figures show that the comparison of mean data using Student–Newman–Keuls and Tukey tests revealed a statistically significant difference among almost all samples obtained, for both untreated and treated samples. The *p*-values calculated by Tukey’s test comparing the untreated samples with the four treated samples corresponded to the critical level of significance. For the average numbers of neutrophils in the connective tissue shell per 1 mm of the length of the implant–organism interface, the *p*-values ranged from 1.58 × 10^−3^ to 1.56 × 10^−2^. For the distribution density of neutrophils per 1 mm^2^ area of the connective tissue shell, the *p*-values ranged from 7.61 × 10^−3^ to 4.94 × 10^−2^.

Two results deviated from these patterns. First, the difference in the specific number of neutrophils in the tissue near the untreated and treated samples with a fluence of 10^15^ ions/cm^2^, calculated by Tukey’s test, was found to be statistically insignificant with a *p*-value of 0.105. Second, the difference in the density of neutrophils for the groups without treatment and with treatment with a fluence of 5 × 10^15^ was also found to be statistically insignificant with a *p*-value of 0.0683. This result is consistent with the Tukey test for multiple paired comparisons of sample means being “softer” than the Student–Newman–Keuls test, and it may “miss” differences in mean data when differences exist.

For neutrophils in the connective tissue shell, no statistically significant differences between the samples treated with different fluences were found at the significance level of α = 0.05. In general, the decrease in the number of neutrophils for the treated samples compared to untreated samples was observed to be statistically significant.

When pairwise comparing the average values of the cellular response to the untreated and treated samples with different doses of ions, the lowest *p*-value was found for the fluence of 10^16^ ions/cm^2^, then in increasing order for the fluence of 5 × 10^14^ ions/cm^2^. The most significant differences were observed for the untreated samples in all quantitative measures of cellular response. For the samples treated with a fluence of 5 × 10^14^ ions/cm^2^, a significant difference in the distribution density of neutrophils was observed. However, for both eosinophils and neutrophils, the statistical analysis of data between different modes did not allow us to reliably indicate the highest-priority treatment fluence.

The thickness of the connective tissue shell formed after 1 and 5 months of the implant being in the organism was analyzed. The ANOVA analysis of variance for the untreated and treated samples showed a difference in mean collagen membrane thickness, with a *p*-value of 9.1 × 10^−3^ for 1 month of exposure and 3.9 × 10^−6^ for 5 months of exposure.

**Figure 12:**
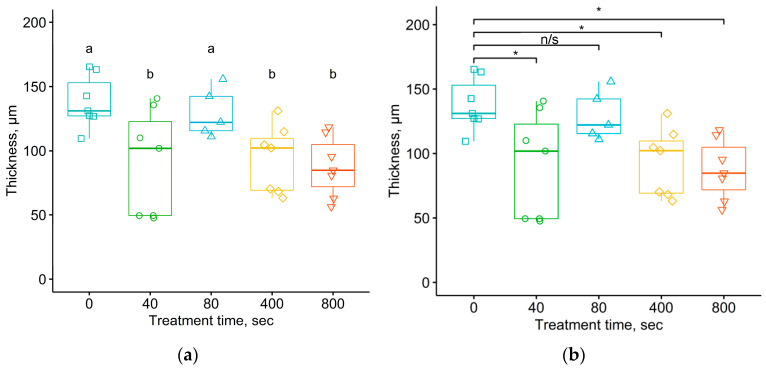
Thickness of the connective tissue shell after 1 month of exposure of the polyurethane implant in the organism under different implant treatment fluences. Comparison of mean values using the Student–Newman–Keuls (**a**) and Tukey (**b**) tests. ANOVA test shows *p* = 0.00909. For Student–Newman–Keuls test, the “a” and “b” notifications are related to the groups of data with significant differences in means between them.

To identify differences between the samples subjected to five different treatment fluences, data analysis was performed using the Student–Newman–Keuls and Tukey tests. For the samples removed from animals after 1 month of exposure, a comparison of sample means using the Student–Newman–Keuls test (Figure 12a) showed that there was a statistically significant difference in the thickness of the connective tissue shell for the group of samples without treatment and all groups of treated samples. The exception was the group of samples treated with a fluence of 10^15^ ions/cm^2^, with *p*-value of 0.985 according to Tukey’s test. It is quite possible that this result was due to the fact that the sample number of this group was smaller than the others due to the loss of an implant with tissue from one of the animals. The *p*-value according to Tukey’s test for only one treatment fluence corresponded to the critical significance level of α = 0.05. In particular, the *p*-value was 0.036 for the fluence of 10^16^ ions/cm^2^ (Figure 12b). There was no significant difference at the significance level of α = 0.05 when comparing the average values of the thickness of the connective tissue shell between samples treated with different fluences.

**Figure 13:**
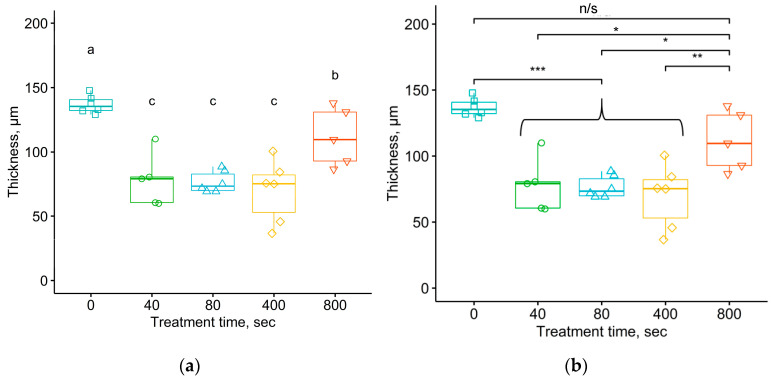
Thickness of the connective tissue shell after 5 months of exposure of the polyurethane implant in the organism under different implant treatment fluences. Comparison of mean values using the Student–Newman–Keuls (**a**) and Tukey (**b**) tests. ANOVA test shows *p* = 3.9 × 10^−6^. For Student–Newman–Keuls test, the “a”, “b”, and “c” notifications are related to the groups of data with significant differences in means between them.

Figure 13 shows the results of the analysis of average thickness of the connective tissue shell using the Student–Newman–Keuls and Tukey method for samples removed from the organism after 5 months of exposure. Both diagrams show that the average thickness of the connective tissue shell near the samples treated with a fluence of 10^16^ ions/cm^2^ was significantly greater than that of samples treated in other fluences, and this value became comparable to the thickness observed for the untreated samples. Interestingly, samples treated with this fluence, after 1 month of exposure in the body, showed a predominance of collagen fibers and fibroblasts over other cellular elements. Perhaps the increase in the thickness of the shell occurred due to the longer duration in the organism. The reason for this histological picture may have been due to the fact that the treatment with a high fluence created a more rigid surface layer with a large number of defects, such as cracks and waves. Probably, this type of the implant surface structure can lead to injury to surrounding tissues, as well as allow proteins and immune cells to penetrate through the resulting cracks into the untreated deep layers of polyurethane and cause a foreign body reaction.

The analysis of the thickness of the connective tissue shell formed after 5 months using the Student–Newman–Keuls and Tukey tests confirmed the statistical significance of the difference in the mean values for the untreated samples and those treated with different fluences, with the exception of the regime with the fluence of 10^16^ ions/cm^2^. When comparing the average thickness values for the untreated samples and those treated with fluences below 10^16^ ions/cm^2^, the significance level of Tukey’s test corresponded to the critical value of α = 0.05 with *p*-values ranging from 1.16 × 10^−5^ to 1.7 × 10^−4^. The significance level of Tukey’s test when comparing the average thickness of the connective tissue shell around the untreated samples and those treated with a fluence of 10^16^ ions/cm^2^ was 0.168. In this case, there was a significant difference in the average values between the data for the samples treated with a fluence of 10^16^ ions/cm^2^ compared to the data for samples treated with lower fluences of ions. The only exception was the result of the comparative analysis of the average thickness of the connective tissue shell by the Tukey method for groups of samples treated with fluences of 10^16^ ions/cm^2^ and 5·10^14^ ions/cm^2^, where the *p*-value was 0.057, slightly greater than the critical significance level of α = 0.05.

Thus, based on the above results, we can conclude that the thickness of the connective tissue shell formed around untreated implants was significantly greater than that of the treated implants. The analysis of the presented statistical data on various characteristics reflecting the cellular response of the organism to the polymer implant allowed us to draw the following conclusions. The difference in the organism’s response between the untreated samples and the treated samples was significant. The difference in the organism’s response between the samples treated with different fluences was insignificant.

## 4. Discussion

Generally, we can consider how a change in the structure of the surface layer of polyurethane as a result of ion treatment affects the body’s immune response. The surface of polyurethane treated with high-energy nitrogen ions is characterized by the presence of condensed aromatic clusters, active free radicals, a high surface energy of 65.4 mJ/m^2^, and a corresponding low water contact angle of up to 37 degrees [49]. In this case, free radicals, like carbon atoms with unpaired valence electrons, are located at the edges of aromatic clusters such as graphene and, due to coupling with the π-electrons of aromatic structures, are stabilized. This allows them to be preserved for quite a long time. Such a surface is chemically highly active and is capable of covalently binding proteins and other molecules in contact with it [49]. The greatest surface activity is observed immediately after treatment. When the treated polymer is exposed to air, nitrogen and oxygen molecules are adsorbed from the air, as well as all molecules of contaminants present in the atmosphere and in packaging materials if the treated polymer comes into contact with their surface. Molecules bind chemically to the surface through reactions with free radicals. As a result, surface activity decreases. However, the ability to covalently attach proteins can persist for quite a long time.

As the treated polymer is stored, the surface energy decreases and the contact angle increases. At the same time, the accumulation of chemically adsorbed molecules and, first of all, atmospheric oxygen occurs. As a result of oxidation, oxygen-containing groups appear in the surface layer of the treated polymer. The concentration of free radicals drops. Such changes depend both on the external environment and on the composition and structure of the treated polymer [49].

The attachment of proteins to the surface layer of the polymer occurs due to the reaction of carbon atoms with unpaired electrons at the edge of the aromatic cluster with the proton donor groups of the protein. As has been shown, a protein molecule can be attached covalently to any amino acid residue. Under optimal conditions for protein application, the surface of the treated polymer is completely covered with a protein layer. Its high surface energy and corresponding hydrophilicity ensure hydrophilic intermolecular interaction with the rest of the protein molecule and do not cause a change in its conformation [36].

This structure of the treated polymer surface causes a change in the response of the cells of the organism’s immune system when the treated implant is inserted into the organism’s tissues compared to the insertion of an untreated polyurethane implant. There is no change in the conformation of adsorbed proteins and, accordingly, the platelets are not activated. The adsorbed proteins are not washed out or replaced due to the Vroman effect (no replacement of firstly adsorbed proteins by other proteins with time). Therefore, no proteins previously in contact with the implant surface are observed in tissues or in the bloodstream near the implant. Accordingly, neutrophils and monocytes are not recruited or differentiated to macrophages. Foreign body giant cells and fibroblasts are not formed. No fibrotic encapsulation or collagen deposition is observed [37,50].

A similar result was observed in a number of our studies on polyurethane treated with high-energy nitrogen ions [35,36,50,51,52]. For the first time, the results showed the absence of a connective tissue shell around the treated SKU-PFL polyurethane after implantation into rats for a period of 4 and 6 months [35]. This was observed from optical micrographs and from the infrared attenuated total reflection spectra of the polyurethane surface. The treatment was carried out on a pulsar ion implanter with 20 keV ions. The water contact angle of the samples was 60 degrees. At the same time, the surface of the treated samples did not come into contact with anything before being introduced into the body. In comparison, the untreated polyurethane showed a normal foreign body response and connective tissue shell formation. Similar results were obtained for periods of 1 month in mice [50,52]. At the time, these results were seen as very revolutionary and were questioned. Therefore, despite the obvious lack of reaction to the foreign body in the experiment, no conclusions about the absence of the foreign body reaction were reached.

The next experiment was carried out with SKU-PFL polyurethane treated in an ILU-4 ion implanter with nitrogen ions with the energy of 20 keV [52]. After treatment, polyurethane samples were stored in plastic bags for 6 months before being implanted into animals. The water wetting angle of the samples immediately before insertion into the organism was 90 degrees. When implanted into the rats, the treated polyurethane showed the usual foreign body response and connective tissue shell formation as for untreated polyurethane.

The next experiment was carried out with PU2363 Pelletane polyurethane [52]. Polyurethane was treated in a plasma immersion ion implanter. Surgery on rats was conducted 9 months after treatment. Some decrease in the foreign body reaction was observed by spectra and microphotos of the polyurethane surface.

The following experiment was conducted with shape memory polyurethane. The polyurethane samples were treated in a plasma immersion ion implanter [51]. Surgery on mice was performed 7 days after the treatment. A decrease in the foreign body reaction was observed using histology and histochemistry methods.

The following experiment was conducted with specially synthesized soft polyurethane. The samples of polyurethane were treated in a plasma immersion ion implanter [50]. Surgery on mice was performed 24 h after treatment. In some samples, an almost complete disappearance of the foreign body reaction was observed using histology and histochemistry methods.

In the present study, a decrease in the thickness of the connective tissue shell near the implant was observed. This decrease was proved by three different methods of statistical evaluation of the measurement results, and was observed for implantation over a short period of 1 month and over a long period of 5 months. There was also a decrease in the concentration of neutrophils and eosinophils in the tissues surrounding the implant. The role of neutrophils in organisms include the destruction of microorganisms after their capture (phagocytosis) or by releasing antimicrobial substances into the intercellular space; the destruction and digestion of damaged cells and tissues; the participation in the regulation of the activity of other cells due to the production of a number of cytokines. The concentration of neutrophils increases in tissues near a wound surface and near foreign bodies that have entered the body. The introduction of an untreated polyurethane implant also causes an increase in the concentration of neutrophils in the tissues surrounding the implant. The appearance of neutrophils in high concentrations is a marker of the body’s immune response to the implantation. However, in the case of a treated implant, the concentration of neutrophils is statistically lower than that of an untreated implant. The functions of eosinophils in body tissues are to carry out the antiparasitic defense of the body, provide the regulation of the immune system by activating and inactivating its mediators, as well as producing a number of inflammatory mediators and cytokines. An increased concentration of eosinophils indicates an inflammatory process in the tissues of the body. The insertion of an untreated implant causes an increase in the concentration of eosinophils in the tissues surrounding the implant. However, near treated implants, the concentration of eosinophils is significantly lower. These results indicate a weakened cellular immune response to the introduction of a treated implant compared to an untreated one. An analysis of other types of immune cells was not performed due to their insufficient number in the tissues around the implant for quantitative assessments.

Similar results of improved biocompatibility were previously obtained for carbon–carbon materials [53,54,55,56,57,58,59,60]. However, the reasons for the improved biocompatibility of such materials remained unclear at the time. Perhaps the improvement in the biocompatibility of such materials is also associated with the presence of free valences in carbon atoms on the surface, at the edges of aromatic clusters, which are formed in the technology of carbon–carbon materials itself. Such free valences in carbon atoms also contribute to the covalent attachment of host proteins from organisms, which then hide the implant from the immune cells, and the immune reaction is not initiated.

The comparison of the results of experiments conducted at different times with different polyurethanes shows that the effect of reducing the foreign body reaction is observed for all materials, regardless of the ion treatment method, such as ion beam implantation or plasma immersion ion implantation. It is observed that there is a visible tendency for the foreign body reaction to decrease with time between the ion beam treatment and the surgery, when the implant comes to contact with organism tissue and proteins. This tendency corresponds to the reasoning surrounding the contribution of free radicals to the activity of the polyurethane surface after treatment. The long-term storage of implants after treatment and the contact of the treated surface with packaging materials and air pollution lead to a decrease in surface activity and a return to the foreign body reaction. More detailed and targeted studies are needed to confirm this assumption.

## 5. Conclusions

The results of the study allow us to definitely state that polyurethane treated with nitrogen ions with 20 keV energy causes a lower cellular foreign body reaction and the formation of a thinner connective tissue shell, compared to untreated polyurethane. In particular, there is a decrease in the concentration of neutrophils and eosinophils in the tissues surrounding the implant. At the same time, the cellular reaction and capsule thickness slightly depend on the ion fluence. Some greater effect of reducing the immune response is achieved when using average treatment fluences around 10^15^ ions/cm^2^. However, this difference is much smaller than that observed when comparing treated implants with untreated implants. The comparison of the obtained results with the results of previous studies with similar polyurethanes and treatment methods allow us to suggest that for the most pronounced disappearance of the foreign body reaction, it is desirable to minimize the time after treatment until the implant is inserted into the organism. This conclusion also corresponds to the surface activity after treatment due to the appearance on the surface of free radicals capable of attaching the organism’s host proteins to the surface of the implant and hiding the implant from the immune cells of the organism.

## Figures and Tables

**Table 1:** Average numbers and densities of eosinophils and neutrophils in the connective tissue near the polyurethane implants after 1 month in the organism per 1 mm length of the implant–organism contour and per 1 mm^2^ area of the shell, respectively.

Fluence, Ions/cm^2^	Eosinophils, Per 1 mm^2^ Area	Eosinophils, Per 1 mm Length	Neutrophils, Per 1 mm2 Area	Neutrophils, Per 1 mm Length
0	31 ± 13	3.8 ± 2.2	22 ± 11	2.8 ± 1.7
5 × 10^14^	5.1 ± 3.7	0.45 ± 0.24	10.7 ± 9.5	0.9 ± 0.4
10^15^	5.2 ± 1.7	0.79 ± 0.11	10.2 ± 3.3	1.54 ± 0.18
5 × 10^15^	6.0 ± 1.8	0.62 ± 0.28	11.3 ± 2.7	1.1 ± 0.4
10^16^	3.4 ± 1.9	0.31 ± 0.18	7.5 ± 2.9	0.69 ± 0.32

**Table 2:** Average thickness of the connective tissue shell around implant samples after 1 and 5 months in the organisms.

Fluence, Ions/cm^2^	1 Month, μm	5 Months, μm
0	133 ± 22	137.0 ± 6.9
5 × 10^14^	91 ± 41	79 ± 22
10^15^	129 ± 19	76.5 ± 8.4
5 × 10^15^	93 ± 26	70 ± 24
10^16^	88 ± 24	112 ± 23

## Data Availability

The original contributions presented in the study are included in the article, further inquiries can be directed to the corresponding author.
